# Enhancing postural control in stroke patients: advances in mechanisms and functional recovery analysis of core stability training

**DOI:** 10.1007/s10072-025-08119-5

**Published:** 2025-04-07

**Authors:** Tingyu Zhang, Jiejiao Zheng

**Affiliations:** 1 Rehabilitation Medicine Center and Institute of Rehabilitation Medicine, West China Hospital, Sichuan University, Chengdu, 610041 China; 2 Key Laboratory of Rehabilitation Medicine in Sichuan Province, West China Hospital, Sichuan University, Chengdu, 610041 China; 3 Department of Rehabilitation Medicine, Huadong Hospital Affiliated to Fudan University, Shanghai, 200040 China

**Keywords:** Core stability training, Stroke, Postural control, Functional recovery, Mechanism

## Abstract

Stroke remains the primary cause of mortality and morbidity in the adult population in China. Postural control dysfunction is a significant and persistent issue commonly observed in stroke patients. Core stability training has been shown to improve postural control in stroke patients, but the accuracy and efficacy of subjective scales used to assess the quality of resulting improvements remain uncertain. The first part of this manuscript reviews the origins and development of core stability training. The second part provides a brief examination of the mechanism by which core stability training affects postural control in post-stroke individuals. The third part reviews the functional recovery outcomes of core stability training as assessed through instrumental gait analysis, with gait spatio-temporal and kinematic parameters enhancing motor control, center of gravity trajectory and kinetic parameters enhancing postural stability, and electromyographic activity parameters enhancing neuromuscular recovery of core muscle groups.

## Introduction

According to data from the Global Burden of Disease, stroke remains the primary cause of mortality and disability among adult individuals in China, and its prevalence continues to increase, with the current number of affected individuals being the highest globally [1]. In addition, despite the progress made in medical advancements that have led to a decrease in stroke mortality rates, a significant number of stroke patients continue to experience motor control dysfunction even after receiving treatment. This dysfunction often manifests as postural control dysfunction, decreased balance, limited mobility, and reduced proprioception [2, 3]. Among the various challenges faced by stroke survivors, postural control dysfunction stands out as a particularly severe and persistent issue, significantly impacting their daily lives [4]. Postural control refers to the capacity to regulate the position of the body in relation to its surroundings, with the aim of attaining stability and orientation. This ability is of utmost importance for the maintenance of body balance and the restoration of walking capability [5, 6]. Therefore, it is imperative to prioritize the investigation and management of postural control disorders in stroke patients within the realm of rehabilitation medicine in China.

In recent years, there has been a growing demand for the rehabilitation of patients with post-stroke hemiplegia. As a result, core stability training (CST) has gained popularity in clinical practice as an effective strategy to improve patients’ trunk control and balance [7, 8]. The term “core” is commonly referred to as the spino-pelvic-acetabular complex [9]. In a broader sense, core stability refers to the capacity to sustain vertebral equilibrium within the boundaries of physiological norms by minimizing displacement caused by disturbances and preserving structural integrity [10].

The enhancement of core stability has been demonstrated to have a significant impact on the preservation of balance, functional mobility, walking capacity, and postural prehension among individuals with post-stroke hemiplegia [11]. Significant evidence from relevant systematic reviews has demonstrated the effectiveness of interventions aimed at improving trunk control and dynamic balance in stroke patients with CST; however, the authors also felt that additional evidence regarding the quality of the enhancement and the effectiveness of the training was necessary [8, 12, 13].

Therefore, this review aims to present a comprehensive analysis of the current progress in CST, its clinical utilization in facilitating functional recovery based on quantitative data such as three-dimensional gait parameters, and to investigate the underlying mechanism of CST’s impact on post-stroke postural control. The objective is to offer valuable insights for future clinical research on post-stroke postural control.

## Methods

This narrative literature review was searched in: Embase, PubMed, Web of Science, CNKI, SinoMed and VIP databases, containing three Chinese and three English databases for a total of six databases. Searches were conducted using the Mesh terms “stroke”, “core stability” and “postural balance” and prioritizing papers from the last five years. The selection included retrospective studies, prospective observational studies, and clinical trials. ClinicalTrials.gov, which refers to clinical trials that have not yet started or for which final results have not yet been published, was also searched in order to provide an up-to-date description of the current state of clinical research in this area, taking into account the aspect of innovativeness. References of the included studies were also screened in order to supplement the narrative. There were no language restrictions on literature searches.

## The origin and development of CST

In 1985, Pope and Panjabi [14] introduced the notion of “spinal stability” to address the issue of lumbar spine instability. The concept of “core stability” was formally introduced by Panjabi in 1992 and subsequently applied to the field of rehabilitation medicine. According to Panjabi [15], the crucial aspect of core stability lies in preserving the neutral stability of the spine, thereby safeguarding the body’s joint functions through a solid structural foundation. The generation of core stability is achieved through the efficient collaboration of the active and passive muscle groups and nerves within the core component during daily activities, ensuring consistent control of the body’s core.

Based on the concept of core stability, CST is gaining recognition in the field of sports injury prevention and management. CST is a movement therapy that is grounded in the principles of the neuromuscular control system. The primary objective of CST is to enhance the strength and stability of the lumbar-pelvic-hip joints as a cohesive unit, thereby promoting the maintenance of the standard anatomical structure of the human body and facilitating the transmission of limb movements. Additionally, CST aims to provide comprehensive reinforcement of both the superficial and deep muscle groups of the human trunk, as well as the movements of the spinal column [16, 17].

In the realm of rehabilitation for stroke patients, the application of core stability training has undergone a gradual evolution. In the early stages, during the late 1990s, when the concept of CST was emerging and being introduced into the field of rehabilitation, CST for stroke patients primarily borrowed from conventional rehabilitation methods. For instance, stroke patients might have been simply instructed to perform some basic core musculature strength training exercises, such as leg raises while lying supine, aimed at initially enhancing the strength of their core musculature and improving their fundamental body control abilities [18]. However, as research progressed into the early 21st century, studies revealed specific abnormalities in muscle activation patterns among stroke patients [19]. Consequently, researchers adjusted the training approaches by incorporating neuromuscular electrical stimulation (NMES) to assist in CST. Yoo et al. [20] indicated that the application of superimposed NMES could result in more effective contractions of the core muscles, with a significant increase in muscle thickness during contractions. From this, stimulating specific nerves can assist stroke patients in activating the correct core musculature, thereby effectively enhancing training outcomes. In recent years, with technological advancements, virtual reality (VR) technology has gradually been integrated into core stability training. Relevant clinical practices have demonstrated that utilizing VR technology creates training environments that simulate daily activities such as walking and climbing stairs for stroke patients, allowing them to engage in core stability training in more immersive and targeted settings [21, 22].

After the widespread adoption of CST, the theory of muscle classification systems has been progressively developed to elucidate the dynamic stability of the core muscle groups in the human trunk [23]. Initial theories classified muscles into two primary categories: deep single-joint muscles responsible for controlling movement and maintaining static stability through centrifugal contraction. These muscles primarily function as local stabilizers, being attached to the vertebrae and their surrounding area. On the other hand, global mobilizers are bi-jointed superficial muscles that generate torque for movement and force through centripetal contraction. These muscles are mainly attached to the torso and limbs [24]. On the basis of their research, Gibbons et al. [25] put forward a functional model that categorized the global mobilizing muscles into two groups: stabilizing muscles (including the internal and external obliques and spinal rami) and mobilizing muscles (such as the rectus abdominis and iliac ribs). Behm et al. [26] have suggested a categorization of global mobilizing muscles into two groups: mobilizing muscles and load-transferring muscles. They argue that load-transferring muscles, which connect the core to the limbs, can operate autonomously but are crucial for maintaining core stability.

In addition, a biomechanical analysis study concluded that there was a significant correlation between core stability and the activation ratio of the internal oblique/rectus abdominis muscles [27]. However, another systematic review reported that among the core muscles associated with physical fitness exercises, the internal obliques had the greatest activity during core stability training, the rectus abdominis, external abdominal obliques, and erector spinae had the greatest activity during free-weight exercise, whereas the lumbar multifidus had the greatest activity during conventional exercises [28].

The theory of muscle classification systems and the application of anatomy and biomechanics of the core stability have gradually reveals the intricacies and distinctions among various muscles, thereby diversifying the utilization of CST and offering novel approaches and concepts for managing patients with musculoskeletal dysfunction in clinical settings.

## Mechanisms of CST in postural control

### Effects of stroke on neural pathways and motor control

Stroke can lead to ischemic or hemorrhagic damage to local brain tissue, directly injuring the motor cortex, basal ganglia, or corticospinal tract, and disrupting signal transmission between the brain and spinal cord. This results in the absence or delay of descending motor control commands, manifesting as delayed activation of trunk muscles, decreased muscle strength, and impaired posture control [2, 29]. For instance, delayed activation of the transversus abdominis muscle can lead to decreased spinal stability, further affecting the coordination of limb movement [18]. On the other hand, abnormalities in sensory pathways after stroke make it difficult for the central nervous system to accurately perceive the spatial position of the trunk and limbs, thereby affecting balance regulation. Consequently, patients often exhibit phenomena such as shifted center of gravity while sitting and asymmetrical trunk posture while walking [30]. In addition, studies have shown that neuronal cell death in the damaged area may be accompanied by synaptic pruning in surrounding areas, inhibiting the formation of new synapses; abnormal compensation (such as excessive use of the healthy side) may also further hinder the recovery of neural pathways on the affected side [31, 32].

### Mechanisms by which CST enhances neuroplasticity

Research indicates that CST, through repetitive and rhythmic actions stimulating the central pattern generator (CPG) in the spinal cord, can facilitate the autonomous activation of intermediate neurons in the spinal cord, bypassing the damaged corticospinal tract, and re-establish motor patterns [33, 34]. For instance, exoskeleton robot training can activate the CPG through precise gait reproduction, promoting neural reorganization at the spinal cord level [34]. Additionally, in unstable support environments, sustained contraction of core musculature can increase the input of proprioceptive signals, stimulating the integrative function of the cerebellum and parietal cortex [35]. This stimulation enhances the brain’s perception of trunk position and movement status, facilitating the reconnection of sensorimotor circuits. Furthermore, CST requires patients to maintain trunk stability during multi-planar movements, which necessitates precise control of core musculature by the motor cortex. A study revealed that CST significantly expands the activation area of the motor cortex, indicating an increase in synaptic density and connectivity strength [36]. Research also suggests that by enhancing trunk symmetry strength, CST can reduce ipsilateral compensation, forcing contralateral muscle groups to participate in movement [37]. This “forced use” mechanism can activate neuroplasticity in the contralateral cerebral hemisphere, promoting functional balance between bilateral hemispheres.

### Mechanisms of postural control regulation and the role of CST

**Fig. 1:**
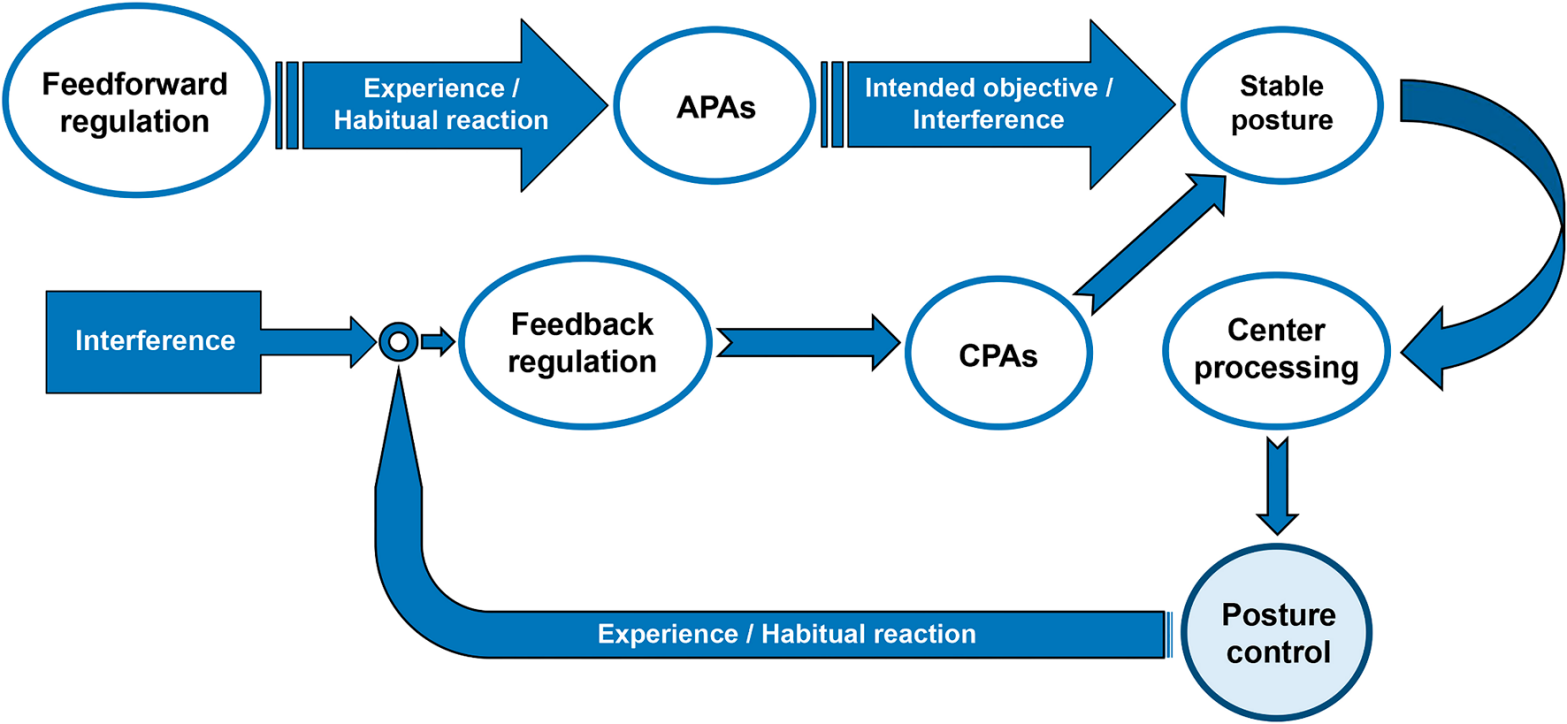
The mechanisms of postural control are finely regulated by the anticipatory postural adjustments (APAs) and compensatory postural adjustments (CPAs)

The stability of the human body’s upright posture has been noted to be unstable. The neuromuscular system is unable to sustain a consistent level of tension due to factors such as heartbeat, fluid flow, and respiration, thereby impeding the body’s ability to attain a state of strict equilibrium. Therefore, the regulation process of postural control is reliant on a continuous maintenance of equilibrium through intricate mechanisms [38, 39]. This intricate mechanism of balance adjustment is linked to both APAs, which are activated by feedforward mechanisms prior to the occurrence of a disturbance, and compensatory postural adjustments (CPAs), which are initiated in response to sensory feedback signals. Before and during the initiation of exercise, APAs and CPAs play a crucial role in achieving postural control. They regulate the center of gravity of the trunk and adjust postural tension to minimize interference with the body [40]. Research has demonstrated that within the classical framework, APAs are considered as an innovative approach to mitigate interference forces generated by dynamic components during pointing activities and to enhance limb movements [41]. The occurrence of APAs is not contingent upon postural stability. The mechanisms of postural control finely regulated by the APAs and CPAs are shown in Fig. 1.

On the other hand, core stability can be defined as a comprehensive activation of the muscles in the trunk, pelvis, and spinal regions, aimed at optimizing coordinated force transmission through core control [42]. It has been proposed that selective recruitment training of the aforementioned muscles helps to reorganize the motor control patterns of the central cortex, thus improving the recruitment patterns of the core muscle groups [10]. Once awareness of volitional contraction and proprioception of the core muscles as the stabilization systems are established, core training to improve neuromuscular control become the priority. Core control plays a crucial role in maintaining postural control. It is primarily regulated by the cerebral cortex, specifically the pontine reticular spinal tract. The advanced APAs of this tract contribute to the restoration of feedforward reflex regulation of the trunk. Consequently, this process enhances postural control in individuals who have experienced a stroke [6, 43]. The primary pathway responsible for modulating postural control is the medial ventral tract, which facilitates downstream motor conduction. Multiple conduction pathways of the medial ventral tract exhibit direct connections with motor neurons that innervate the proximal muscles of the neck, back, and limbs. As a result, these pathways play a crucial role in postural control stabilization and coordination of limb movement [44].

## Functional recovery of the CST assessed through instrumental gait analysis

CST has regained attention in the realm of physical therapy and sports rehabilitation in recent years. This renewed interest is attributed to its potential to compensate for the inadequate training of trunk and core muscles in conventional movement therapies. Additionally, CST offers a training approach for individuals with postural control dysfunction following a stroke. Recent systematic reviews have demonstrated that the incorporation of pertinent randomized controlled trials (RCTs) that utilize balance, walking, and functional assessment as outcome measures has led to notable enhancements in CST [8, 12, 45–47]. However, as early as the 1960s, researchers proposed that proximal stabilization is essential for distal activity [48]. It was suggested that maintaining proper stability in the trunk can enhance postural control and movement performance. Hence, individuals who have experienced a stroke may enhance their functional activity through the utilization of compensatory strategies. It is important to note that improvements in subjective scale scores may not accurately depict the actual level of postural control in stroke patients [49, 50]. To discern between the proper recuperation and compensation of patients’ activity patterns, as well as to explore the effects of CST on lower limb motor performance, investigating information pertaining to motor injuries and functional recovery from the standpoint of quantitative data on three-dimensional gait parameters.

### Enhancing motor control through the utilization of gait Spatiotemporal and kinematic parameters

Patients who experience post-stroke postural control dysfunction often exhibit kinematic characteristics, including an abnormal postural swing spectrum, abnormal weight distribution, reduced four-point synchronization, and an increased risk of falling [51]. At all three roundtable discussions on stroke recovery and rehabilitation, it was emphasized that kinetic and kinematic indicators should be prioritized for assessing the restoration of patients’ motor quality [52–54]. It was further suggested that changes in these parameters offer a more accurate indication of functional improvement in patients. Therefore, investigating the impact of CST on motor control in stroke patients, specifically in relation to spatio-temporal and kinematic parameters, holds significant research significance.

Survivors of post-stroke hemiplegia commonly exhibit atypical walking patterns, such as scissor gait and circle gait, as a result of central nervous system impairment. These deviations in gait are evident in the temporal and spatial parameters of stroke patients’ walking, as stated in previous research [55]. Therefore, it is imperative to monitor gait parameters in order to evaluate the patient’s motor and balance capabilities. In the RCT conducted by Bai et al. [56], a balance meter was employed to gather data on weight distribution index and general stability. Additionally, a three-dimensional gait analysis system was utilized to analyze gait parameters in order to assess the impact of CST on the balance function of individuals who have experienced a stroke. The findings indicated that the weight distribution index of the affected limbs in the patient group, as well as the stability coefficients and various parameters related to walking such as cadence, speed, and stride length, exhibited statistically significant improvements. These improvements were found to be superior to those observed in the control group that underwent conventional rehabilitation training. Xiao et al. [57] conducted a study on hemiplegic patients and determined that the primary gait characteristics observed were a decrease in gait speed and an increase in the step length symmetry index (affected/healthy side step length). The researchers utilized the Helen Hayes marker set model [58] for gait analysis. The study revealed that the CST had a significant positive impact on improving gait speed and restoring stride symmetry in hemiplegic patients who had experienced a stroke. Fan et al. [59] conducted a study in which they employed an omnidirectional intensive motor training system, along with a space suit, as the primary tool for early rehabilitation of elderly stroke patients. They also utilized CST either in combination with the motor training system or as a standalone intervention. The study findings indicated that both the combined application of the two interventions and the use of CST alone resulted in improved gait spatiotemporal parameters, joint motion parameters of the hips, knees, and ankles, as well as the double support phase in the gait time parameter. Zhou et al. [60] demonstrated significant improvements in cadence, speed, and stride length ratio in three-dimensional gait among stroke patients by utilizing a combination of balancing needles and CST.

In a study conducted by Olczak [61], an observational approach was employed to investigate the impact of core stability on coordinated movement parameters of the trunk and lower extremities in stroke patients. The findings revealed that core stability plays a significant role in facilitating more accurate movement of the trunk in both the coronal and sagittal planes. Additionally, it was observed that core stability contributes to increased foot elevation from the ground, improved gait, and enhanced range of movement in stroke patients. Based on our findings, it can be inferred that alterations in gait parameters have the potential to serve as a more perceptible indicator of postural control dysfunction in individuals with post-stroke conditions. This enhanced visibility enables clinical therapists to accurately identify the specific dysfunction and subsequently tailor interventions to yield optimal outcomes.

### Enhancement of postural stability through improvement of center of gravity trajectory and kinetic parameters

The assessment of postural stability and balance can be effectively conducted by analyzing the trajectory of the center of human gravity, which is considered one of the fundamental approaches [62]. Li et al. [63] conducted a study to investigate the impact of combining CST with extracorporeal shockwave therapy on postural control in stroke patients. The results of their study revealed that CST with visual feedback yielded superior outcomes in terms of reducing the length of the center of gravity trajectory, increasing the ratio of trajectory overlap, minimizing disparities in the distribution of body mass in the anterior, posterior, left, or right regions, as well as improving muscle tone in the calf triceps muscle and enhancing ankle joint mobility. Pilkar et al. [64] employed a novel core-strengthening apparatus called AllCore360° to administer CST interventions on three stroke patients. The researchers documented alterations in the patients’ center of gravity through postural tracings. The results revealed a decrease in coronal center of gravity excursion in all patients, with one patient experiencing a substantial reduction of 69%. Van Criekinge et al. [65] demonstrated that CST resulted in improvements in various gait parameters in stroke patients. Specifically, CST increased step length, speed, and sagittal center of gravity displacement, while also enhancing mobility and reducing step width and coronal center of gravity displacement. However, the study did not find any significant differences in lower limb kinematics. Additionally, the authors combined their findings with a previous study that showed CST improved trunk and gait control as well as biomechanically-based walking processes [66]. Notably, this study is significant as it is the first to demonstrate the normalization of trunk kinematics in patients following trunk training while walking.

Furthermore, Zhou et al. [60] demonstrated that the utilization of balancing needles in conjunction with CST resulted in a significant reduction in both the area of the center of gravity trajectory and the length of swing in patients. This effect was observed in individuals who performed the task with their eyes open for 60 s and with their eyes closed for 10 s. Gao et al. [67] conducted a study comparing the total length of the center-of-pressure trajectory and the area of the center-of-pressure ellipse in stroke patients using the Zebris balance tester. The results indicated that the improvement in balance function due to CST was evident in the redistribution of gravity in the lower extremities. This redistribution included both the right and left limbs, as well as the affected limb in the plantar aspect of the affected foot. It has been concluded that the implementation of CST can effectively rationalize the distribution of plantar pressure in patients. Sheng [68] employed the identical balance tester and observed similar findings, indicating a general reduction in the total length of the center of pressure trajectory, the area of the central ellipse, the transverse diameter of the central ellipse, and the longitudinal meridian of the patient. Meanwhile, Liu et al. [69] employed VR in conjunction with CST and also observed a significant improvement in the elliptical area of the patients’ center of pressure. The aforementioned findings indicate that evaluating alterations in the center of gravity’s trajectory enables clinical therapists to promptly assess the patient’s condition, make timely adjustments to the content and intensity of rehabilitation training, and ensure the patient’s bilateral muscle strength is well-balanced.

Furthermore, De Luca et al. [70] employed an innovative robotic apparatus to conduct CST in order to investigate alterations in dynamic stability and trunk control among individuals who have suffered from stroke. The findings indicated that the patients’ dynamic balance in the plane of instability, as well as their dynamic balance in the plane of resistance to interference, exhibited improvement. The participants exhibited improved trunk control without the need for compensatory strategies, and these improvements were sustained for a period exceeding three months. The patients demonstrated a decrease in the utilization of compensatory strategies in their daily lives following the completion of the intervention. Zhang [71] conducted a study to examine the weight distribution index of stroke patients with hemiplegia during eye-open and eye-closed conditions using the Tetrax balance instrument. The findings indicated that CST demonstrated superior efficacy compared to double lower limb vibration training, with immediate effects being more pronounced. Haruyama et al. [72] discovered that CST enhanced the active mobility of pelvic tilt in the sagittal plane among stroke patients. This improvement can be utilized in conjunction with gait parameters and center of gravity trajectory evaluation to assess the kinematic parameters of CST.

### Neuromuscular recovery of core muscle groups based on electromyographic activity parameters

Post-stroke hemiparesis occurs when there is a disruption or absence of motor signals from the motor cortex to the spinal motor neurons. The restoration of spinal innervation is crucial for the neural foundation of motor control [73]. Electromyography (EMG) data, utilized for electrical diagnosis and detection, is intricately linked to the transmission of neuroelectric signals from the spinal cord to the muscles [74]. At the same time, the EMG data can provide insights into the extent to which the activation of motor units is impaired in stroke patients, as well as the underlying mechanisms contributing to motor dysfunction [75]. Thus, EMG encompasses valuable neural data pertaining to the performance of motor tasks in individuals affected by stroke, warranting comprehensive investigation.

It has been demonstrated in previous studies that the electromyographic activity of core muscle groups, including the rectus abdominis, external abdominal obliques, latissimus dorsi, and erector spinae, can serve as an indicator of neuromuscular control dysfunction in patients with poststroke postural control dysfunction [76]. Based on the aforementioned study, Zhou [77] employed CST under suspension to examine the balance function of individuals who had suffered from stroke. The surface EMG signal detection data collected prior to treatment revealed notable variations in integrated EMG, median frequency, and mean power frequency solely in the multifidus muscles, while no significant differences were observed in the rectus abdominis, external abdominal obliques, or erector spinae muscles between the healthy and affected sides. The findings of the study demonstrated that the implementation of CST while the patient was under suspension effectively mitigated the disparity observed in the polydactyl muscle data between the healthy and affected sides. Consequently, this intervention facilitated a more equitable distribution of workload between the two sides, promoting a balanced muscular effort. Another study conducted by Zhou et al. [60] examined the effects of balancing acupuncture combined with CST on stroke patients. Surface EMG tests were performed on the quadriceps, tibialis anterior, and gastrocnemius muscles. The results showed a significant increase in the integrated EMG of all three muscle groups, indicating improved neuromuscular recovery. Additionally, the root mean square amplitude was significantly decreased, suggesting enhanced muscle strength. These findings demonstrate the potential of balancing acupuncture combined with CST in facilitating neuromuscular recovery and improving muscle strength in stroke patients.

On the other hand, Lee et al. [78] conducted a study to measure the timing of activation of anticipatory postural adjustments (APAs) in patients with post-stroke postural control dysfunction during rapid forward shoulder flexion. The researchers used EMG to assess the activation of bilateral external abdominal obliques, erector spinae, transversus abdominis, and internal abdominal obliques. The results showed that conventional CST treatment led to a reduction in the time required for APAs activation in all groups. Additionally, dynamic neuromuscular stabilization-based CST achieved even faster activation of APAs. Yoon et al. [79] discovered that dynamic neuromuscular stabilization-based CST resulted in a greater increase in median EMG amplitude values of the transversus abdominis and intra-abdominal obliques in stroke patients with hemiparesis compared to CST based on neurodevelopmental therapy. Additionally, their research showcased that CST can yield improvements in core stability through the utilization of a compression biofeedback device, as well as increases in the values of transversus abdominis muscle thickness as measured by ultrasound imaging.

In addition, Pilkar et al. [64] conducted a study where they gathered EMG data on the rectus abdominis, latissimus dorsi, and upper erector spinae muscles bilaterally in patients during a follow-up period. The results of the study indicated that there was considerable variation in the neuromuscular responses of the participants, particularly in the rectus abdominis muscle. They reached the conclusion that while there was increased activation observed in certain muscles (rectus abdominis, right latissimus dorsi, and right upper erector spinae), it was not possible to draw definitive conclusions due to the absence of normative maximum voluntary isometric contractions.

### Functional recovery in conjunction with other indicators

In addition to the quantitative data obtained from the analysis of three-dimensional gait parameters, certain studies have employed ultrasound measurements of core muscle thickness in stroke patients as an observational indicator. Four RCTs [80–83] utilized CST, CST with ultrasound visual feedback, and CST based on dynamic neuromuscular stabilization. All interventions observed resulted in an increase in the thickness of the transversus abdominis muscle on the affected side, while Aycicek et al. [84] observed an improvement in the thickness of the multifidus muscle. Yoon et al. [83] proposed that the implementation of dynamic neuromuscular stabilization, in conjunction with the transversus abdominis muscle, resulted in an increase in the thickness of the diaphragm and the affected internal abdominal oblique muscle. The RCT conducted by Wang [85] demonstrated that CST resulted in an increase in diaphragmatic mobility during calm inspiration and at the end of maximal inspiration. However, it did not have a significant impact on diaphragmatic thickness. However, Du et al. [82] discovered that among stroke patients experiencing deep sensory dysfunction, only CST with ultrasound visual feedback demonstrated a positive impact on the thickness of the transversus abdominis muscle on the affected side. This finding suggests that the observed improvement may be attributed to the compensation of sensory input dysfunction through the visual pathway.

In conclusion, it has been found that CST has the potential to enhance three-dimensional gait and joint movements by utilizing kinematic parameters. Additionally, CST has been shown to decrease the displacement of the center of gravity trajectory and promote equal weight distribution in the lower limbs of individuals suffering from post-stroke postural control disorders. Meanwhile, the EMG data indicated that core and lower limb muscle neuromuscular recovery could be improved through the use of CST. Additionally, the utilization of ultrasound measurements revealed that CST resulted in an increase in the thickness of the core muscles. Furthermore, it was observed that CST also enhanced and equalized core strength on both the healthy and affected sides.

## Conclusion

Based on the aforementioned findings, it can be inferred that the implementation of CST should prioritize the patient’s capacity to regulate trunk and limb movements. This approach enables the coordination and stabilization of diverse muscle groups, resulting in the progressive enhancement of accurate muscle control in the management of posture. Consequently, the patient’s motor system becomes synchronized, leading to the development of standardized and balanced movement patterns in daily tasks. Ultimately, the implementation of this intervention enhances the individual’s overall quality of life and promotes a smooth and successful reintegration into society.

As an emerging exercise therapy technique, the majority of clinical studies conducted on CST have demonstrated positive outcomes. On the other hand, due to multifaceted functional problems in conjunction with other therapeutic modalities has been progressively rising, owing to the complex functional issues in the body that accompany postural control dysfunction in stroke patients. The effectiveness of CST in clinical practice has been demonstrated through multiple research studies, emphasizing its significance.

However, the clinical application of CST poses numerous challenges. The global community continues to grapple with the ongoing challenge of successfully translating clinical research findings into real-world clinical settings. Reports suggest that only a small fraction of research findings possess the capacity to substantially influence clinical practice, and it frequently requires several years for these findings to catalyze changes in the field [86]. On the other hand, the prioritization of conventional physical therapy over CST as a primary exercise therapy can be attributed to the intuitive sensations experienced by patients during conventional physical therapy, such as the gradual restoration of muscle strength, pain reduction, and increased walking time. This preference is observed in many hospitals and among patients, despite their awareness of the documented benefits of CST over conventional physical therapy as demonstrated in relevant studies.

Most notably, the field of clinical practice is currently lacking a comprehensive and specialized standard methodology for CST. Based on our review, it was observed that the majority of the methodological sections in the studies included utilized a generalized approach to overall core training. Nevertheless, there is a dearth of scholarly literature addressing the subject of specialized training that focuses on the development of specific core muscles. What we anticipate is to elucidate the specific attributes of impaired function in each patient, in accordance with the guidance provided by pertinent guidelines, as well as to explore the discrepancies between motor function and rehabilitation goals. Subsequently, our objective is to execute personalized treatment strategies that are specifically designed to cater to the distinctive attributes of each individual. This initiative seeks to not only establish a standardized approach to the clinical application of CST, but also to generate financial resources for its ongoing clinical research.

In addition, our research findings indicate that only one study administered a subsequent evaluation three months post initial assessment, with limited information available on the enduring impacts. Moreover, there exists a dearth of scholarly literature delving into the specific pathways by which CST impacts postural stability in individuals recovering from strokes, with several cited studies possessing lower-impact. Consequently, there is a pressing requirement for additional in-depth inquiries to scrutinize this domain comprehensively and advance our comprehension.

Future research should prioritize the standardization of the method employed for the application of CST. The future trajectory of research entails the application of a methodical and specialized CST approach for individuals suffering from postural control disorders arising from diverse clinical neurological conditions. This approach is integrated with a variety of therapeutic modalities in order to attain targeted and long-lasting improvements.
